# Risk Management During the Transition From Hospital to Home: A Multiple Case Study Documenting the Experience of Patients Living With a Major Neurocognitive Disorder, Their Caregivers, and Healthcare Professionals

**DOI:** 10.1177/23743735241259553

**Published:** 2024-08-05

**Authors:** Véronique Provencher, Chantal Viscogliosi, Julie Lacerte, Monia D’Amours, Didier Mailhot-Bisson, Lise Gagnon, Guy Lacombe

**Affiliations:** 1 School of Rehabilitation, 7321 Université de Sherbrooke, Sherbrooke, Canada; 2 Research Centre on Aging, Sherbrooke, Canada; 3 School of Nursing, 7321 Université de Sherbrooke, Sherbrooke, Canada; 4 Psychology Department, 7321 Université de Sherbrooke, Sherbrooke, Canada; 5 Department of Medicine, 7321 Université de Sherbrooke, Sherbrooke, Canada

**Keywords:** healthcare transitions, risk management, major neurocognitive disorder, patient experience

## Abstract

Understanding the risks in the months following hospital discharge is crucial for healthcare professionals to ensure the need for assistance is met. However, this may be challenging in the case of patients living with a major neurocognitive disorder (PLMNCD). Thus, it is important to incorporate patients’ and caregivers’ experiences of the transition from hospital to home in the risk assessment. This multiple case study comprised 7 PLMNCD, their caregivers, and occupational therapists. Fifty-four interviews, conducted just before, as well as 3 weeks and 3 to 6 months after hospital discharge, were qualitatively analyzed. Results revealed that risk management during the hospital-to-home transition is a dynamic process aimed at establishing a satisfactory routine while avoiding adverse events. This risk management process, which identifies challenges over time and between stakeholders, involves (a) determining the seriousness and acceptability of risks, (b) reflecting on ways to manage risks, and (c) taking steps to manage risks. This knowledge will help to provide more appropriate care and services that strike a balance between safety and autonomy.

## Introduction

Patients living with a major neurocognitive disorder (PLMNCD) have a high rate of potentially avoidable rehospitalizations.^1,2^ Therefore, it is important upon discharge to help them and their caregivers manage their physical and psychological risks in the months following their return home. Identifying risks upon discharge of PLMNCD is, however, a major challenge for healthcare professionals (HPs).^3,4^ In the assessment process, incorporating patients’ and caregivers’ experiences of the hospital-to-home transition may shed light on potential risks that could occur over a 24-h period (eg, nighttime agitation) and in the following months. It may also help to understand the refusal of home services as PLMNCD are known to have a greater tolerance of risks than their caregivers and HPs.^5^

Some studies have documented the experience of both PLMNCD and their caregivers^6,7^ as well as that of both HPs and caregivers^8,9^ while transitioning from hospital to home. Poole et al^10^ explored the perspectives of PLMNCD, caregivers, and HPs during this critical period concerning relocation decisions. To our knowledge, no other studies have investigated how psychological and physical risks are viewed by these stakeholders (patients, caregivers, and HPs) and how they are managed together before and for a few months after discharge. Having greater knowledge of the experience of these stakeholders upon discharge will help provide better care and prevent avoidable adverse events postdischarge.

This study thus aimed to understand the experiences of PLMNCD, their caregivers, and HPs surrounding the prevention of adverse events during the transition from hospital to home. More specifically, our goal was to explore the processes used to manage risks (how and why) before and shortly after discharge and in the weeks following the return home.

## Methods

### Design and Participants

A qualitative multiple case study^11^ was designed to understand risk management among PLMNCD during the transition from hospital to home. Participants were recruited in two short-term geriatric units (Sherbrooke, Canada, a country with universal health coverage). Each of the 10 cases comprised: (a) an early-stage PLMNCD; (b) their primary caregiver; (c) the occupational therapist (OT) who evaluated the patient in the hospital; and (d) the OT who evaluated the patient at home. OTs were selected as the HP since the care team often relies on their expertise to assess risks related to daily activities.^12^ This number of cases was found to be suitable for gathering sufficiently diverse viewpoints (PLMNCD, caregivers, and HPs).^13^ To be eligible, patients had to: (a) have a major neurocognitive disorder (MNCD; Alzheimer's disease or mixed dementia) at mild to moderate stages according to DSM-V criteria; (b) be discharged to their previous house, apartment, or seniors’ residence; (c) be assessed at hospital and home within 6 weeks postdischarge; and (d) demonstrate the ability to consent to research (Nova Scotia criteria^14^). Participants were excluded if they displayed behaviors (aggression and confusion) that could interfere with study participation.

### Data Collection and Measures

Sixty-four semistructured interviews were conducted by one of the coauthors [CV] with extensive expertise using the qualitative approach and 25 years of experience working with PLMNCD. Individual interviews took place in the hospital before discharge and at home ∼6 weeks after discharge with each patient, caregiver, and HP. Patients and caregivers were also interviewed 3 to 6 months postdischarge.

The pretested interview guide, including 12 questions for patients and caregivers and 23 for HPs, aimed to document their perceptions in order to understand: (a) type, severity, and acceptability of risks, especially when adverse events occur and (b) care and services to be recommended to manage these risks. To promote information recall and reduce reluctance to talk about sensitive topics, patients, and caregivers were asked to “talk about their typical day,” that is, what they did from morning to night. To explore patients’ perspectives without requiring them to acknowledge their cognitive deficits, a case scenario presented a fictional older adult experiencing memory difficulties. Sociodemographic and health data were collected and information from field notes shed light on some contextual elements during the analysis.

### Data Analysis

Interview transcripts were analyzed using qualitative content analysis, which is a comprehensive and suitable approach for case study research.^15^ The coding grid, comprising themes derived from the literature and induced from the data, focused on risk perception, risk management strategies, acceptability of risk and management strategies, and issues surrounding the transition. An intracase analysis was first performed, including a comparison of measurement times and stakeholder perspectives. A cross-case analysis was then done to identify common themes and subthemes, pinpoint variations, and develop overarching themes.

To ensure rigor, a research assistant trained in qualitative research [JL] performed the analysis using NVivo software (version 12). Written summaries using tables, diagrams, and extracts from transcripts were discussed with the principal investigator [VP] at three key stages in the process, namely after an in-depth analysis of the first case, half of the cases, and the dataset. Her solid expertise in risk assessment of frail older adults and discharge planning helped to identify aspects to be analyzed in more depth and confirm the anchoring of emerging themes. One of the coresearchers [DMB] with extensive experience in qualitative research coanalyzed the data and covalidated all the themes that emerged. The person who collected the data [CV] also contributed to the initial coding of the first interviews and the validation of the results. Her in-depth knowledge of the participants and incorporation of her field notes helped to contextualize the data. Members of the research team who collected or analyzed the data had no ties to the participants.

## Results

### Case Descriptions

Table 1 shows the characteristics of the patients, caregivers, and OTs included in each case. Three of the 10 cases could not be considered in the analysis because of death, relocation, and dropout after T1. One patient died after T2, but the data collected at T1 and T2 were retained in the analysis. Ultimately, 54 interviews from 7 cases were analyzed.

**Table 1.** Participants’ Characteristics in Each Case of the Study.

Participants	Characteristics	Cases									
		1	2	3	4	5	6	7	8	9	10
Patients	Gender	Male	Female	Female	Female	Female	Female	Female	Male	Female	Female
	Age	85	81	86	84	86	92	77	92	92	85
	Type of dwelling	Apartment	Apartment	Private house	Seniors’ residence	Private house	Seniors’ residence	Private house	Apartment	Private house	Private house
	Living situation	With wife	Alone	Alone	Alone	Alone	Alone	T1 alone/T2 with sister	Alone	Alone	With husband
	Reasons of hospitalisation	Elective surgery	Cognitive assessment	Post fall fracture	Post fall fracture	Fall, loss of autonomy	Baker cyst rupture	Fall, loss of autonomy	Back pain, falls	Post fall fracture	Cognitive assessment
	Cognitive diagnostic	MNCD—mixed	MNCD	MNCD—Alzheimer	MNCD—vascular	MNCD—mixed	MNCD	MNCD—mixed	MNCD—mixed	Not assigned	MNCD—Alzheimer
	Comorbidities	ASCD, TCI, CABG	Bradycardia, AHT	AHT, CRT	FSBD, AHT, ASCD	AHT, VI, osteoporosis	AHT, FSBD, osteoporosis	FPS, MD	AHT, ASCD, osteoporosis	Diabetes	FSBD
Primary caregivers	Age	69	—	62	37	61	68	75	—	66	87
	Link with patient	Wife	Son	Son	Residence's director (F)	Daughter	Daughter	Sister	Son	Daughter	Husband
Occupational therapists (OT)	T1 (hospital)	OT 1	OT 1	OT 1	OT 1	OT 2	OT 2	OT 1	OT 1	OT 2	OT 1
	T2 (community)	OT 3	OT 3	OT 3	OT 3	— (death)	— (relocation)	OT 3	OT 3	OT 4	— (drop out)

*Cognitive diagnosis*—MNCD: major neurocognitive disorder.

*Comorbidities*—AHT: arterial hypertension; ASCD: atherosclerosing coronary disease; CABG: coronary artery bypass grafting; CRT: chronic renal failure; FPS: fall post syncope; FSBD: fall syndrome with balance disorder; GSTU: geriatric short-term unit; HGU: hospital geriatric unit; MD: macular degeneration; MNCD: major neurocognitive disorder; PPN: peripheral polyneuropathy; TCI: transient cerebral ischemia; VI: venous insufficiency.

F: female. – Information not provided by the person; – – T2/T3 not carried out (deceased, relocated, and dropped outpatients not considered in the analysis).

OT 1: female, 25 years in profession, 24 years within organization; OT 2: female, 2 years in profession, 6 years within organization; OT 3: female, 25 years in profession and within organization; OT 4: female, 1 year in profession and within organization.

### Risk Management During Transition: A Dynamic Process

Throughout their experience with MNCD, patients, caregivers, and OTs strive to establish a satisfactory routine while avoiding adverse events. Their risk management process involves: (a) determining the seriousness and acceptability of risks, (b) reflecting on ways to manage risks, and (c) taking steps to manage risks. All stakeholders perform these various steps at their own pace and at different times during their transition. Table 2 gives examples of participants’ quotations related to each theme, subtheme, and code resulting from the qualitative analysis.

**Table 2.** Qualitative Analysis—Examples of Quotations Related to Each Theme and Code.

Themes	Codes	Examples of quotations
**Risk management during transition: A dynamic process**		
Determining the seriousness and acceptability of risks	Consideration of the person's abilities and past habits	In the kitchen, everything that was automatic, she washed it all, cleaned it all, everything. It's easy to do automatically, so I think that with a routine, at home […], the risks will be less. (OT-hospital, Case 2, T1)
	Perceived fit between the person's needs and the support in place	I found that the risks are still… limited, in the sense that her son is quite mobilized, and she seems to be… relatively in a routine. […] She also seems to automatically call her son or her friend when there's a problem. So I thought they [the risks] were minor. (OT-community, Case 2, T2)
	Consistency with the person's values and benefits for well-being and maintenance of a positive identity	Her two sisters, they told me she would prefer to manage the risk rather than live in a glass bubble. They believe that freedom is more valuable than security. I think that in this sense, staying in her home makes sense. (OT-community, Case 7, T2)
	Probability of serious adverse events makes the risk unacceptable.	But I just want to say that when you found someone like that once [on the floor, unconscious], after that, security becomes a priority. (Caregiver, Case 7, T1)
	Increase in the unacceptability of risk when judgment is affected	His refusals would be based more on: “I don’t need it, I can still do it.” […] The fact is he doesn’t see, he doesn’t realize it. […] He thinks he doesn’t have any cognitive problems so he doesn’t accept the diagnosis. […] In his mind, it's not a true diagnosis. (OT-hospital, Case 1, T1)
Reflecting on ways to manage risks	Prior experiences, such as wish to avoid having strangers in their home, make families reluctant to accept services.	And I don’t see myself having a stranger in the house, you know what’d happen… I’d be so embarrassed, I wouldn’t know what to do with someone. (Caregiver, Case 1, T3)
	Importance of considering how the person feels and giving them time to accept and feel the need	We are letting things happen so he can make his decision when he's ready… He must feel unable to meet his own needs. They will offer him a place in a residential long-term care center (CHSLD). In any case, it must come from him. (Caregiver, Case 8, T3)
Taking steps to manage risks	Strategies are based on changing behaviors, modifying how things are done, or adapting to the environment.	We take precautions to avoid falling. [INTERVIEWER: So, your precautions, do you know what they are?] It's how to walk. [So you walk slower?] Yep. (Patient, Case 7, T3)
		At one point, I thought the stairs were going to be a problem but not at all. Well, I say “not at all” but, you know, she has to hold on, she holds on very well but she has to hold on. (Caregiver, Case 7, T3)
		No, she didn’t fall again. No. [INTERVIEWER: Okay. There was a big improvement anyway.] Yes. Yes, anyway. [INTERVIEWER: How do you explain this improvement?] I think she uses her walker more. I think she takes less chances. (Caregiver, Case 4, T3)
	Some tend to avoid stressful situations, for example, by giving up risky activities.	I’ll tell you: I had my car and when I gave it up, it was me who gave it up. I was driving along and I said: “Where am I going?” I pulled over near the sidewalk and thought about it, and then, I found it. So I knew inside that it had happened, that it was time to stop driving. I stopped […] because I said, I could hit someone and I could kill myself. (Patient, Case 4, T1)
	Caregivers rely on healthcare providers to understand the causes of MNCD and the diagnosis, or to decide what steps to take to resolve conflicts and access services.	As for his driver's license, the children didn’t really agree that he should have it. They would have liked him to give it up. That annoyed him so we didn’t push it. I said to myself: “Let them [the doctors] do that—they’ll see him over there [at the hospital]. […] I spoke to the doctor about it. Then I spoke to Dr S [otorhinolaryngologist] […] And… afterwards I spoke to the cardiologist, and all three said he was okay to drive.” (Caregiver, Case 1, T3)
	Relocation is the last option to consider.	We are quite clear that if her place is here [in her own home] and that's what she wants, we’ll do everything to ensure she stays here, you understand? We’ll do everything. (Caregiver, Case 7, T3)
**Risk management: Challenges over time and between stakeholders**		
Dealing with uncertainty over time	Risk situation is often perceived as unstable, fluctuating from day to day. Its causes and evolution are difficult to determine.	I saw a big improvement since she left the hospital because she wants to stay at home, she can reason and she has a fantastic memory. […] In any case, I look forward to them doing another assessment because maybe it’ll be more precise what she is suffering from. (Caregiver, Case 7, T2) But she is surprising because some days like… when I’m not there, I come back and […] I find out that she went to the post office. (Caregiver, Case 7, T3)
	Patients and caregivers may anticipate adverse events but remain uncertain about the evolution of cognitive or physical impairments and the ability to stay at home.	At the hospital, they didn’t want to let her leave because her diabetes wasn’t stabilized and all that. I returned to look after her for two weeks and that's when they said: “Okay, we’ll let her leave.” (Caregiver, Case 9, T1) I think she came back like before […] but she was more fragile. […] She's not worried but she's more careful because she says: “I don’t want to go back to the hospital.” […] She is in the process of regaining her strength and resuming her little routine. (Caregiver, Case 9, T2) That doesn’t concern me at all. I say to myself: she can fall again like we all can. She is careful, she is more fragile, however. It doesn’t concern me because the CLSC carefully monitors […] her diabetes. That's what had caused her fall, low blood sugar. […] She doesn’t have them anymore [dips in her sugar level]. (Caregiver, Case 9, T3)
Dealing with new relationship issues: Maintaining integrity despite dependence	Growing dependence after discharge creates the need to review the roles of family members towards each other.	So (…) he [Patient 1] says to the children: “I’d like you to say why you don’t want me to babysit your kids, and that I can look after your kids.” Okay… so the children made him understand, with sensitivity, that he's deaf, he can’t hear… and he doesn’t walk! So if one of the kids falls, how will he manage to go… you know (Caregiver, Case 1, T3)
	Challenge for patients to preserve their autonomy and self-esteem when faced with the need for increased assistance	When I was a girl, I organized things without help and I helped other people a lot. I helped but I had difficulty letting people help me. I’m now starting [to accept help]. […] Because I can see that my health is declining. (Patient, Case 4, T1)
	Caregivers feel more responsible for patients’ well-being and safety as their cognitive health declines.	I told her [Patient 7]: “As soon as I realize—I will go there regularly [to see her at home]—that your safety is or may be jeopardized, we will review our decision [concerning staying at home].” I was very frank. I have her mandate and I feel responsible in that regard. (Caregiver, Case 7, T1)
	Risk of caregivers’ burnout due to constant monitoring of risks affecting their loved one	So we [caregiver and his sister], we are overtaxed. We say: “We can’t do it any more.” We can’t do more than we’re doing, we don’t want to do more than we’re doing. (Caregiver, Case 8, T2)
	Conflict with life plans	I spent my life working […] I’ll go and see my mother. I really love my mother… But I can’t be there 100% and ruin my retirement… (Caregiver, Case 2, T1)
	Tensions between family members caused by differing opinions	If it was too risky, I’d see it… […]. You know, it's like I told you, the children are exaggerating because… Me and him [Patient 1], we don’t see anything… complicated, you know. (Caregiver, Case 1, T3)
		After I confronted him with his situation […] he started to consider me one of the bad guys. (Caregiver, Case 8, T3)
	Potential for developing a harmonious partnership	It's lucky he's here [her son living with her]. I rely on him. […] There aren’t too many risks because he thinks about them [medications]. […] It's good for him too, being here. He doesn’t pay any rent, nothing. (Patient, Case 3, T2)
		We agreed [main caregiver and her sister]. I said: “Me, [sister's name], I’ll be there when you’re not there, and we’ll get organized.” (Caregiver, Case 7, T2)
	Caregivers’ and healthcare providers’ dilemma of prioritizing protection over self-determination	Sure, he always does what he wants. We want him to have some autonomy, so we put him in a situation where he is supposed to be autonomous. […] But I notice that he has a lot less autonomy than he says. (Caregiver, Case 8, T2)
		There was a series of… things [adverse events] so, should I be concerned? About the decisions he makes, because he can be impulsive and it's not adapted. […] He can't see himself going. (Caregiver, Case 8, T3)

Abbreviations: MNCD, major neurocognitive disorder; OT, occupational therapist.

***Determining the Seriousness and Acceptability of Risks***. To appraise risks, stakeholders consider not only the seriousness of potential adverse events but also the person's abilities and past habits when trying to predict risk occurrence. Risks are considered to be properly managed when stakeholders believe that the support in place meets the person's evolving needs.

Moreover, risk-taking tends to be acceptable when it is consistent with the person's usual way of managing risk and when it appears beneficial for the person's well-being and maintenance of a positive identity. Conversely, risks become unacceptable when serious adverse events seem very likely to occur or to have occurred already (eg, traumatic accident). This is especially true when the person's judgment is seriously questioned. Missing pieces of information, such as events that are forgotten or denied, which is common in dementia, can lead to a biased appraisal of risk, which influences the whole risk management process.

***Reflecting on Ways to Manage Risks***. The reluctance of patients and caregivers to seek the care and services recommended to help them manage risks depends greatly on prior experiences. Faced with the limited availability of homecare services, some patients and caregivers said they were used to managing risks themselves and did not want strangers in their home.

In addition to the objective benefits derived from the service provided (eg, having chores done by someone else), how the PLMNCD and their caregivers feel when receiving services has a major impact on their acceptance or refusal. Their need to make their own decisions and protect an identity rooted in their independence also affects their receptiveness when homecare is recommended. To foster the acceptance of services and avoid confrontation, some caregivers and HPs reported facilitating meaningful dialogue between stakeholders to find more acceptable solutions, emphasizing the benefits of the services for their loved ones and giving the PLMNCD time to come to terms with the need for assistance.

***Taking Steps to Manage Risks***. Many patients prefer a combination of human assistance and equipment to manage risks related to motor disabilities and cognitive deficits. Formal care and services as well as “spontaneous/intuitive” strategies are used by patients and caregivers to engage in meaningful activities and connect with others. While some of these strategies are based on changing behaviors (self-regulating or being assertive), modifying how things are done, or adapting to the environment, other strategies seek to avoid stressful situations, especially by giving up risky activities. Caregivers tend to rely on healthcare providers to understand the causes of MNCD and the diagnosis or to decide what measures to take to resolve conflicts (eg, about driving) or access services. For all stakeholders in this study, relocation was the last option considered.

### Risk Management: Challenges Over Time and Between Stakeholders

In their risk management processes, stakeholders must address various challenges, including dealing with uncertainty over time and the emergence of new relationship issues.

***Dealing With Uncertainty Over Time***. Risk management during the transition from hospital to home requires patients and caregivers to continuously adjust to an ever-changing reality. Due to the degenerative nature of the disease and acute problems leading to hospitalization, the risk situation is often perceived as unstable, fluctuating from day to day. Its causes and evolution are difficult to determine.

After discharge, patients’ and caregivers’ expectations are tested as they return to their daily reality, with either a better-than-expected recovery or new difficulties that require them to change their routine. In their efforts to manage risks, patients and caregivers may anticipate adverse events but remain uncertain about the evolution of cognitive or physical impairments and the person's ability to remain at home.

***Dealing With New Relationship Issues: Maintaining Integrity Despite Dependence***. Risk management in the context of growing dependence after discharge creates new relationship issues and the need to review the roles of various family members in relation to each other and, more generally, society. When faced with the need for increased assistance from caregivers and friends, one of the main challenges reported by patients is how to preserve their autonomy and self-esteem. Being identified as an “ill person” is opposed to the desire to be perceived as a “valuable person.”

For caregivers, their loved ones’ declining cognitive health makes them feel progressively more responsible for their well-being and safety. At the same time, the constant monitoring of risks and associated tasks puts caregivers at risk of burnout and might conflict with their life plans. Diverging perspectives concerning the risk situation can also create tensions between family members or with the person with cognitive deficits. Conversely, reorganizing their roles can also lead to a harmonious partnership between them.

Finally, as cognitive impairments may begin to lessen people's ability to take care of themselves or make decisions, caregivers and HPs may be faced with the dilemma of whether or not to prioritize safety over people's autonomy.

## Discussion

This study investigated the experience of PLMNCD, their caregivers, and their HP throughout the transition from hospital to home. Our findings revealed that risk management during this transition is a dynamic process that attempts to maintain a precarious balance to avoid adverse events. This process includes various challenges related to changes over time and interaction between stakeholders.

Risks and assistance tend to be more acceptable to all stakeholders when they align with past habits and when the positive consequences of risks outweigh the negative. Our results suggest that the acceptability of risks and assistance is linked to lifestyle, and more broadly to the person's values, self-esteem, and identity. Our findings thus underline the importance of documenting the person's perspective to help achieve a balance in risk management by considering their values and life story and supporting the expression of their needs.^16,17^ “Spontaneous” strategies, consistent with the person's life story, family dynamics, and the caregiver's habits, seem to make the risks more acceptable. Since there is a dearth of resources and delays in organizing homecare after discharge,^18,19^ valuing these strategies may avoid the need to seek “external” services to support aging in place.

The process of accepting risks and assistance seems to be linked to a “cost–benefit” risk analysis as recommendations are more readily accepted when the perceived benefits outweigh the disadvantages. More specifically, some people may refuse needed services because they feel uncomfortable having “strangers” in their home or because of the perceived relationship benefits of being helped by a loved one. For others, refusing help from a relative might be consistent with the value of “doing it alone,” even if tasks are performed less efficiently and in a riskier way. These findings are in line with positive risk-taking theories,^20,21^ which may lead to a paradigm shift by incorporating a shared risk decision-making approach in routine care.

Our results also highlight caregivers’ difficulty finding a balance between safety and autonomy after discharge. The challenge is how to legitimize risk-taking while minimizing serious consequences that may harm not only the person but also the caregiver (eg, feeling of guilt). How should one deal with differences of opinion when the caregiver's needs and wishes (eg, to rest) conflict with the person's preferences (eg, desire to be cared for by someone familiar)? Since caregivers of PLMNCD have a high risk of psychological morbidity, depression, stress, and anxiety,^22,23^ they may become unable to continue providing support after hospitalization.^24,25^ It is then crucial to initiate a dialogue with them about the support they will be able and/or ready to offer during the weeks following discharge, to avoid relocation and support quality of life. Since older adults and caregivers do not all have the same risk tolerance, the use of patient-centered decision and communication support tools fostering positive and responsible risk management may facilitate discussions searching for acceptable interventions.^20^ Our results also suggest that while changes in care roles between family members may exacerbate tensions, they may also lead to a positive partnership. A recent study confirms the importance of understanding family dynamics to better support families in their “reorganization,” as they achieve balance and value themselves in their new roles.^26^

Our longitudinal results reflect the difficulty of dealing with uncertainty as well as relationship issues. Caregivers expressed the need to be supported by HP in promoting the acceptability of certain interventions and avoiding conflicts. Caregivers thought that recommendations that are “harder to accept” (eg, concerning driving) should be proffered by an HP, who is seen as a neutral person and authority figure, rather than giving them this responsibility, which may harm their relationship with their loved one. HPs also face communication challenges during the process regarding what they should or should not say, especially upon discharge. In line with Hestevik et al,^27^ many patients and caregivers reported feeling overwhelmed during the hospitalization and they may not be able to remember or understand all of the recommendations or emotionally deal with some information about the diagnosis. Some recommendations need to be shared before discharge while others make more sense to patients and caregivers once they see how things are going at home. To achieve a balance between providing information without increasing anxiety or discouragement, an open discussion may be initiated with the patient and caregiver regarding potential issues to expect during these transitions and whether their own strategies might be useful in overcoming them. Some simple new technology^28,29^ may also provide timely information to act upstream of challenges that may arise after discharge and refer them to the “right resource” at the “right time” to avoid rehospitalization or relocation.

Our findings suggest it is essential to understand the patient's view of “risky situations,” including perceived links between the situation (risks) and the consequences, in order to provide them with acceptable assistance.^10^ Interestingly, our participants were often able to take a critical look at at-risk situations not involving them, which suggests that self-criticism is affected while connections people make between risks and their consequences seem preserved. The use of case scenarios may help to identify strategies acceptable to some patients based on their recognition of safety issues in “other patients.” More research is needed to determine whether less self-criticism in MNCD might be triggered by mechanisms aimed at preserving self-esteem and integrity. Moreover, in a world where MNCD is still stigmatized, fear of being judged unconsciously influences the acceptability of the disease and, more broadly, of interventions. Promoting community-based interventions, such as MNCD-friendly communities, might help manage threats to the person's moral integrity.

### Limitations

This study has some potential limitations. First, there are possible biases due to memory recall and social desirability as some older adults may have feared being relocated if they acknowledged being at risk. However, using “typical day” and “case scenario” during the interviews should have minimized this potential limitation. Second, the transferability of our results to other contexts should be viewed with caution, given recruitment and mortality challenges, and the lack of information on cultural background and literacy level. However, our study included participants from two different units, which increased its external validity.

## Conclusion

Despite the promotion of patient-centered care, this is one of the few studies to document the perspective of PLMNCD longitudinally throughout their transition in care. By documenting individual values using interview guides and case scenarios, this study sought to look beyond physical risks and uncover emotional risks, especially relationship conflicts with family members and the fear of being judged by others. Future studies could explore how family history and social stigma may affect the acceptability of recommendations to support risk management from a lifespan and societal perspective.
